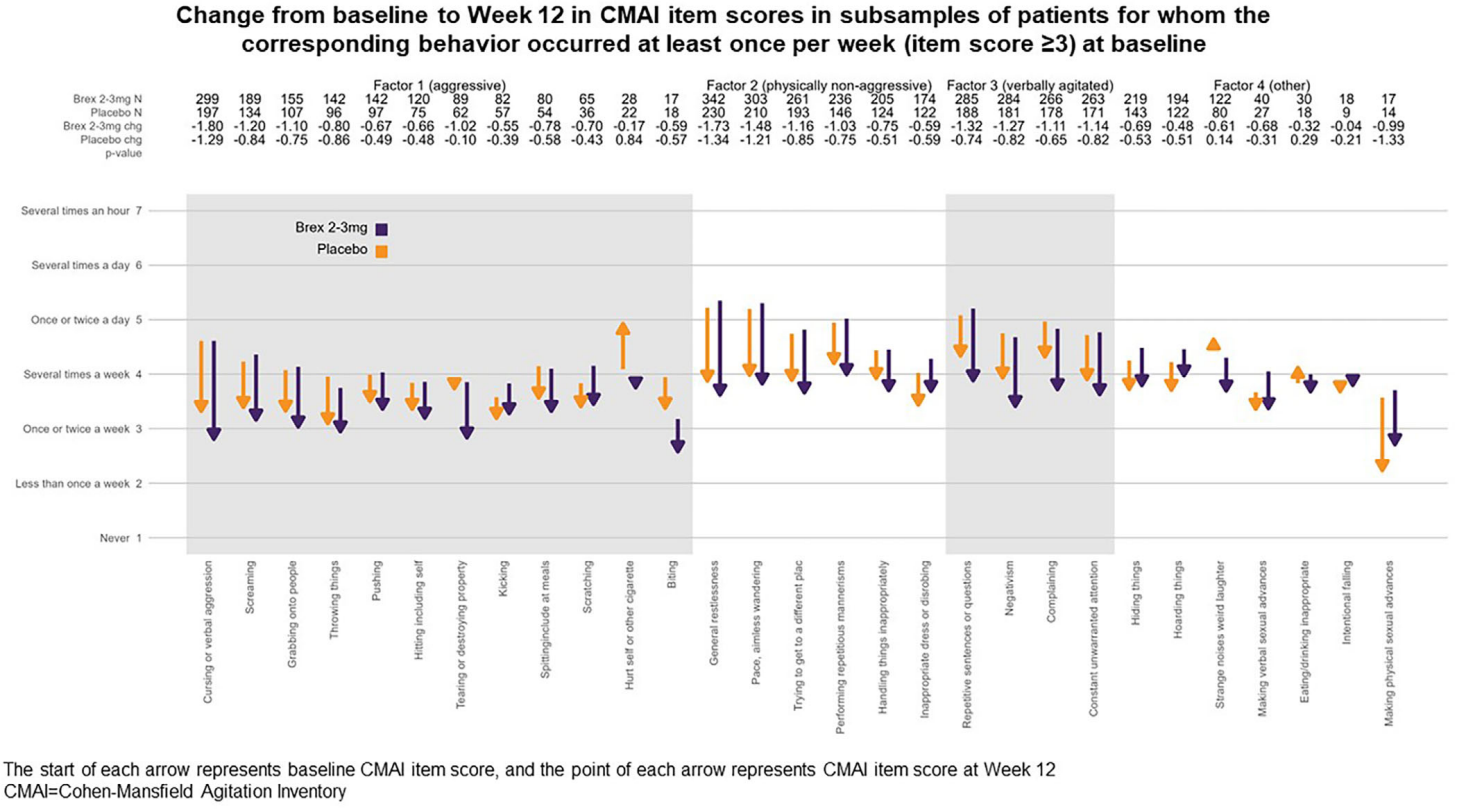# Efficacy of brexpiprazole on frequently occurring agitation behaviors in patients with dementia due to Alzheimer’s disease: *post hoc* pooled analysis of two randomized controlled trials

**DOI:** 10.1002/alz.092174

**Published:** 2025-01-03

**Authors:** Malaak Brubaker, David Wang, Sanjeda R Chumki, Pedro Such, Zhen Zhang, Anton M Palma

**Affiliations:** ^1^ Otsuka Pharmaceutical Development & Commercialization Inc., Princeton, NJ USA; ^2^ Lundbeck LLC, Deerfield, IL USA; ^3^ H. Lundbeck A/S, Valby, Copenhagen Denmark

## Abstract

**Background:**

Patients with dementia due to Alzheimer’s disease may experience multiple different agitation symptoms – including excessive motor activity, verbal aggression, and physical aggression – at varying frequencies. The efficacy of brexpiprazole 2 or 3 mg/day on 29 individual agitation behaviors (Cohen‐Mansfield Agitation Inventory [CMAI] items) was previously evaluated. Building upon that work, this *post hoc* analysis aimed to determine the efficacy of brexpiprazole on the same individual agitation behaviors, but specifically focusing on those patients who were frequently experiencing the behaviors at baseline.

**Method:**

Data were included from two 12‐week, randomized, double‐blind, placebo‐controlled, parallel‐arm trials of fixed‐dose brexpiprazole in patients with agitation associated with dementia due to Alzheimer’s disease (ClinicalTrials.gov identifiers: NCT01862640, NCT03548584). Efficacy was assessed using the CMAI, which measures the frequency of occurrence of 29 agitation behaviors (each scored 1 [never] to 7 [a few times an hour]). In this analysis, least squares mean change from baseline to Week 12 in each CMAI item score was determined in subsamples of patients for whom the corresponding behavior occurred at least once per week (item score ≥3) at baseline. Data for brexpiprazole 2 or 3 mg/day (FDA‐approved dose) and for placebo were pooled.

**Result:**

Data were analyzed for 610 patients (brexpiprazole, n = 363; placebo, n = 247). At baseline, the mean (standard deviation) number of different agitation behaviors occurring at least weekly was 12.8 (4.3) per patient. The agitation behaviors most commonly occurring at least weekly were general restlessness (n = 572), pacing/aimless wandering (n = 513), and cursing or verbal aggression (n = 496). For some behaviors (e.g., intentional falling, n = 27), conclusions were limited by small subsample sizes. Across the CMAI items, least squares mean score changes from baseline to Week 12 ranged from ‐1.80 to ‐0.04 for brexpiprazole, and ‐1.34 to +0.84 for placebo, with a greater numerical reduction for brexpiprazole versus placebo on 24/29 items (Figure).

**Conclusion:**

Patients with agitation associated with dementia due to Alzheimer’s disease exhibited many different frequently occurring agitation behaviors. Fixed‐dose brexpiprazole 2 or 3 mg/day was associated with a numerically greater reduction in the frequency of most of these frequently occurring agitation behaviors versus placebo.